# Illicit Anabolic Steroid Use and Cardiovascular Status in Men and Women

**DOI:** 10.1001/jamanetworkopen.2025.26636

**Published:** 2025-08-29

**Authors:** Laust Frisenberg Buhl, Louise Lehmann Christensen, Rikke Hjortebjerg, Selma Hasific, Clara Hjerrild, Stefan Harders, Mads Lillevang-Johansen, Dorte Glintborg, Marianne S. Andersen, Mario Thevis, Caroline Kistorp, Jon Jarløv Rasmussen, Jes S. Lindholt, Axel C.P. Diederichsen, Jan Frystyk

**Affiliations:** 1 Department of Endocrinology, Odense University Hospital. Odense, Denmark; 2 OPEN Patient Data Explorative Network (OPEN), Odense University Hospital, Region of Southern Denmark, Odense, Denmark; 3 Department of Clinical Research, Faculty of Health Sciences, University of Southern Denmark, Odense, Denmark; 4 Steno Diabetes Center Odense, Odense University Hospital, Odense, Denmark; 5 Department of Cardiology, Odense University Hospital, Odense, Denmark; 6 Department of Radiology, Odense University Hospital, Odense, Denmark; 7 Centre for Preventive Doping Research, Institute of Biochemistry, German Sport University, Cologne, Germany; 8 Department of Nephrology and Endocrinology, Rigshospitalet, University Hospital of Copenhagen, Rigshospitalet, Denmark; 9 Department of Clinical Medicine, Faculty of Health and Medical Sciences, University of Denmark, Copenhagen, Denmark; 10 Department of Internal Medicine, Endocrinology Unit, Holbæk Hospital, Holbæk, Denmark; 11 Department of Vascular Surgery, Odense University Hospital, Odense, Denmark

## Abstract

**Importance:**

The illicit use of anabolic androgenic steroids (AAS) is common among recreational athletes, yet studies on adverse cardiovascular outcomes, especially in female AAS users, are sparse.

**Objective:**

To assess cardiovascular status in Danish male and female recreational athletes using AAS compared with not using AAS.

**Design, Setting, and Participants:**

This cross-sectional study in a single center in Denmark included recreational athletes aged 18 years or older who were active AAS users, previous users (defined as those who discontinued AAS use at least 3 months prior to the study), or nonusers, all recruited from fitness centers between March and December 2022.

**Exposure:**

Cumulative lifetime use of AAS.

**Main Outcomes and Measures:**

Presence of atherosclerosis (carotid, femoral, and coronary artery plaques) and cardiac function assessed by echocardiography. Linear regression was used to estimate regression coefficients for echocardiographic characteristics and logistic regression to estimate odds ratios (ORs) for carotid and femoral plaques, coronary artery calcium (CAC) scores, and coronary noncalcified plaques (NCPs).

**Results:**

Of 164 participants, 80 (48.8%) were active AAS users (median age, 35 [IQR, 30-43] years; 61 men [76.2%]), 26 (15.9%) were previous users (median age, 36 [IQR, 28-51] years; 18 men [69.2%]), and 58 (35.4%) were nonusers (median age, 40 [IQR, 31-46] years; 42 men [72.4%]). Median cumulative lifetime AAS use was 2.2 (IQR, 1.2-7.2) years for active and 2.2 (IQR, 1.0-5.5) years for previous users. No group differences were observed when comparing the number of participants with femoral (active users, 15 [19.7%]; previous users, 5 [19.2%]; nonusers, 11 [20.8%]; *P* = .89) or carotid (active users, 24 [31.2%]; previous users, 12 [46.2%]; nonusers, 13 [24.1%]; *P* = .47) artery plaques or CAC scores (median score was 0 across all groups with range of 0-228 for active users, 0-800 for previous users, and 0-163 for nonusers; *P* = .36), whereas a statistically significant difference in the prevalence of coronary NCPs was found between active users (19 [23.8%]) and nonusers (6 [10.3%]) (*P* = .03). However, in confounder-adjusted logistic regression, longer cumulative lifetime AAS use was associated with higher odds of a positive CAC score (OR, 1.23; 95% CI, 1.09-1.39; *P* = .001) and presence of coronary NCPs (OR, 1.17; 95% CI, 1.05-1.30; *P* = .004). AAS use exceeding 5 years was associated with greater severity of calcifications (n = 94; χ^2^ = 9.78; *P* = .04). Echocardiography showed that cumulative AAS use was associated with worse left ventricular (regression coefficient: 0.08; 95% CI, 0.03-0.12; *P* = .002) and right ventricular (0.08; 95% CI, 0.03-0.13; *P* = .001) global longitudinal strain. Nearly all athletes (35 of 36) with more than 5 years of cumulative AAS use had ventricular mass greater than and left ventricular ejection fraction below the median of the normal range.

**Conclusions and Relevance:**

In this cross-sectional study, cumulative lifetime AAS exposure was associated with adverse cardiovascular findings and impaired ventricular function in both sexes, and athletes with AAS exposure exceeding 5 years showed more severe calcification. The findings support measures to prevent AAS use by both men and women in recreational sports.

## Key Points

**Question:**

Is illicit use of anabolic androgenic steroids (AAS) associated with cardiovascular status in male and female recreational athletes?

**Findings:**

In this cross-sectional study of 164 participants (80 active AAS users, 26 previous users, and 58 nonusers), coronary artery calcification and echocardiographic measures were associated with cumulative lifetime exposure to AAS in both sexes. Findings were most pronounced for participants with more than 5 years of AAS intake.

**Meaning:**

Cumulative lifetime AAS exposure was an independent factor associated with coronary atherosclerosis and myocardial dysfunction in both sexes, suggesting preventive measures against use of AAS in recreational sports should target both men and women.

## Teaser

This cross-sectional study examines associations between use of illicit anabolic androgen steroids and cardiovascular outcomes including coronary atherosclerosis in adult male and female recreational athletes in Denmark.

## Introduction

The use of androgenic anabolic steroids (AAS) is prevalent among recreational athletes, with an estimated lifetime prevalence of 3.3% in men and 1.6% in women.^1,2,3,4^ This is concerning because long-term AAS intake has been associated with numerous detrimental health outcomes, particularly cardiovascular complications^5^ and premature death.^6^

The association between AAS use and coronary artery disease (CAD) emerged from case reports and small observational studies of male athletes, which reported severe premature CAD,^7,8,9^ myocardial infarction,^10,11^ and sudden cardiac death^12,13,14^ in younger male AAS-using bodybuilders and weightlifters. Over the past decade, larger studies^9,15,16,17^ have substantiated these findings, reporting a positive association between coronary artery plaque volume and the duration of AAS use.^15^ Nevertheless, to our knowledge there are no available data on peripheral artery disease in athletes using AAS.

Beyond CAD, postmortem examinations,^18,19,20^ echocardiographic assessments,^15,16,21,22,23,24,25,26,27^ and cardiac magnetic resonance imaging^28,29^ have indicated the presence of AAS-related cardiomyopathy characterized by left ventricular (LV) remodeling or hypertrophy. This has been associated with declines in LV systolic and diastolic function^15,16,22,23,24,25,26,27,28,29^ as well as right ventricular (RV) systolic function.^30^

Our knowledge regarding long-term cardiovascular effects of AAS use in recreational athletes derives primarily from studies in men, although female athletes also use AAS illicitly.^3^ Therefore, we investigated cardiovascular status in a population of Danish male and female recreational athletes using AAS and compared the findings with those from recreational athletes not using AAS. The primary objective was to assess peripheral artery plaque formation through vascular ultrasonography of the carotid and femoral arteries, coronary artery calcification (CAC), and coronary noncalcified plaques (NCPs) using coronary computed tomography (CT) angiography and cardiac status by echocardiography.

## Methods

### Recruitment

The Fitness Doping in Denmark (FIDO-DK) Study is a nationwide cross-sectional cohort study. In the current cross-sectional study, we used FIDO-DK data from Odense University Hospital (OUH), Denmark. This study was approved by the Region of Southern Denmark regional ethics committee. Participants provided written informed consent. The reporting follows the Strengthening the Reporting of Observational Studies in Epidemiology (STROBE) reporting guideline. The aim was to examine adult recreational male and female athletes aged 18 years or older who had used AAS illicitly for at least 3 months. After enrolling AAS users, we recruited a control group of healthy nonusers of similar sex and age who engaged in strength training at least twice a week. Participants were grouped as active AAS users if they reported current use of AAS or had discontinued the use of AAS within 3 months prior to the study. Participants grouped as previous users had discontinued AAS use at least 3 months before the study. Nonusers had never used AAS. A detailed study protocol has been published recently.^31^ Participants were recruited from fitness centers between March and December 2022.

### Study Program

Participants reported medical history, use of prescribed medications, supplement use, alcohol and tobacco consumption, recreational drug use, physical activity regimen, and socioeconomic status. Subsequently, participants filled out questionnaires about fitness training history and AAS experience, including use status (current or previous), time intervals between intake, types of AAS used, dosage, duration of use, age at onset of use, maximum weekly dose, and cumulative lifetime AAS use. Information also included the illicit use of other performance-enhancing drugs and recreational drugs, as previously outlined.^31^

Urine samples were tested for steroid metabolites (Centre for Preventive Doping Research at the German Sport University Cologne) to verify or dispute the reported AAS status. Guidelines for identifying exogenous androgens included the detection of synthetic testosterone compounds and nonsteroidal anabolic agents in urine samples and a testosterone-epitestosterone ratio greater than 4.^32^ Blood samples were collected to measure routine biochemistry, performed by the Department of Biochemistry at OUH. In addition, we measured height, weight, waist and hip circumference, body mass index (BMI, calculated as weight in kilograms divided by height in meters squared), hand grip strength (Jamar hand dynamometer), and body composition by bioimpedance (SOZO Digital Health Platform).

All participants underwent a bilateral B-mode vascular ultrasonography examination of the carotid and femoral arteries, focusing on the presence of plaques, using an ultrasonography device (Logiq E 12 MHz linear transducer, GE HealthCare). Images were digitally stored and subsequently analyzed. Plaques were defined as focal structures that extended into the arterial lumen by at least 0.5 mm or had thickness 50% greater than the surrounding intima media thickness, or an intima media thickness greater than 1.5 mm. Plaque findings were evaluated by an external validator blinded to AAS status.

Subsequently, transthoracic echocardiography (Vivid E95, GE HealthCare) was performed. Recordings were saved and analyzed using EchoPAC software (GE HealthCare) by an external operator blinded to AAS status. We recorded cardiac dimensions, LV and RV systolic function, LV diastolic function, and left atrial volume. Systolic function was evaluated using the Simpson biplane method assessing LV ejection fraction (LVEF) and global longitudinal strain (GLS) analyses. Diastolic function was evaluated by peak mitral inflow velocity (E), early septal and lateral LV relaxation velocity (E′), and left atrial volume.

We then applied coronary CT angiography (Force CT scanner, Siemens Healthineers) coupled with a semiautomatic computer program (syngo.via, Siemens Healthineers) to estimate CAC using the Agatston method.^33^ Coronary NCPs were visually assessed by an experienced cardiologist (A.C.P.D.) who was blinded to AAS status. Coronary NCPs were defined as coronary arterial wall lesions that exhibited low attenuation values relative to the surrounding tissues.^31^

### Statistical Analysis

Continuous variables were presented as median (IQR or range) and categorical variables as number (percentage). Normality of data distribution was assessed using quantile-quantile plots and the Shapiro-Wilk test. To address nonnormality of the data distributions, transformations were applied.

Group comparisons were performed using 1-way analysis of variance followed by Tukey honestly significant difference for post hoc analysis or the Kruskal-Wallis test followed by the Dunn test with Holm-Šídák adjustment for multiple comparisons, as appropriate. Post hoc tests were performed only when overall group differences were statistically significant. Tests for trends across ordered groups were performed using regression analyses or the Jonckheere-Terpstra test. χ^2^ tests were used for categorical variables. Assumption of homogeneity of variances was tested using the Levene test.

The outcomes of cumulative lifetime duration of AAS intake were assessed by regression models. Linear regression was used to estimate regression coefficients and associated 95% CIs for echocardiographic characteristics, and logistic regression was used to estimate odds ratios (ORs) and 95% CIs for carotid and femoral plaques, CAC scores, and coronary NCPs. The CAC variable was dichotomized (score of 0 vs >0). Multivariable regression models were adjusted for age, sex, body fat percentage, hours of hard exercise and strength training per week, family history of coronary disease, blood pressure, total cholesterol, tobacco smoking status, alcohol consumption, and use of recreational drugs. Multivariable normal imputation methods were applied to address missing categorical covariables and maximize power. Missing categorical variables (weekly hours of exercise and strength training, family history of coronary disease, tobacco smoking status, and alcohol consumption) were imputed on a continuous scale and rounded to the nearest integer. The imputed models were validated by comparing the mean (SD) of the imputed dataset with the original dataset. Missing data did not exceed 5.0% for any imputed variables. All statistical tests were 2-sided, and *P* values less than .05 were considered significant. Statistical analyses were performed using Stata, version 18 (StataCorp LLC).

## Results

### Cohort Characteristics

The study included 164 participants (106 [64.6%] of whom had used AAS): 80 (48.8%) were active AAS users (61 men [76.2%], 19 women [23.8%]; median age, 35 years [IQR, 30-43 years]), 26 (15.9%) were previous users (18 men [69.2%], 8 women [30.8%]; median age, 36 years [IQR, 28-51 years]), and 58 (35.4%) were healthy nonusers (42 men [72.4%], 16 women [27.6%]; median age, 40 years [IQR, 31-46 years]) (Table 1). Sex was self-reported. No group differences in age or sex distribution were observed (Table 1). Across sexes, active AAS users had the highest BMI, lowest body fat percentages, and highest muscle percentages compared with previous users and nonusers (all between-group differences were significant) (Table 1).

**Table 1.** Characteristics of the Participants in the Entire Cohort, Stratified by AAS Use

Variable	Participants^a^				*P *value		
	Active AAS users (n = 80)	Previous AAS users (n = 26)	Nonusers (n = 58)	*P* value for group comparison	Active AAS users vs previous users	Active AAS users vs nonusers	Previous AAS users vs nonusers
Sex							
Female	19 (23.8)	8 (30.8)	16 (27.6)	.75	NA	NA	NA
Male	61 (76.2)	18 (69.2)	42 (72.4)				
Age, y	35 (30-43)	36 (28-51)	40 (31-46)	.14	NA	NA	NA
Men	33 (28-39)	35 (28-49)	43 (31-50)	.02	.21	.005	.41
Women	44 (33-47)	39 (32-53)	36 (32-43)	.21			
Height, m	1.78 (1.71-1.84)	1.75 (1.69-1.81)	1.82 (1.75-1.86)	.02	.41	.02	.01
Men	1.81 (1.75-1.86)	1.79 (1.72-1.83)	1.83 (1.82-1.88)	.006	.26	.01	.004
Women	1.65 (1.61-1.71)	1.70 (1.61-1.75)	1.73 (1.69-1.75)	.04	.30	.01	.28
Body surface area, m^2^	2.10 (1.92-2.29)	1.99 (1.87-2.15)	2.07 (1.93-2.14)	.14	NA	NA	NA
Men	2.17 (2.06-2.33)	2.06 (1.93-2.17)	2.11 (2.05-2.24)	.10	NA	NA	NA
Women	1.81 (1.73-1.92)	1.79 (1.72-1.92)	1.84 (1.74-1.93)	.81			
BMI	29.0 (26.4-31.4)	26.5 (25.0-28.6)	25.2 (23.4-27.9)	<.001	.006	<.001	.13
Men	29.4 (27.7-32.2)	27.4 (25.6-29.9)	26.5 (24.0-28.3)	<.001	.03	<.001	.09
Women	25.9 (24.9-29.1)	24.6 (22.9-26.3)	24.3 (22.9-25.5)	.04	.13	.01	.59
Body fat percentage	14.8 (11.1-17.2)	18.2 (14.2-22.2)	21.4 (17.7-23.6)	<.001	.001	<.001	.06
Men	13.1 (10.4-16.5)	17.8 (12.3-21.2)	20.0 (16.3-22.5)	<.001	.004	<.001	.06
Women	16.5 (15.9-21.6)	20.2 (16.7-26.9)	25.5 (22.1-27.9)	.006	.13	.002	.26
Muscle percentage	41.3 (38.4-43.1)	38.4 (34.1-41.1)	37.2 (33.9-40.0)	<.001	.01	<.001	.25
Men	41.7 (39.8-43.6)	40.0 (37.8-42.7)	38.7 (36.3-40.4)	<.001	.09	<.001	.07
Women	36.6 (31.9-41.5)	33.8 (30.4-34.5)	32.2 (29.8-34.9)	.007	.04	.003	.68
Blood pressure, mm Hg							
Systolic	138 (129-147)	123 (110-131)	132 (125-139)	<.001	<.001	.03	<.001
Men	138 (130-148)	129 (122-134)	135 (126-141)	.002	<.001	.03	.06
Women	135 (116-143)	107 (104-113)	128 (121- 126)	<.001	<.001	.75	<.001
Diastolic	77 (71-84)	72 (68-75)	78 (68-83)	.02	.007	.33	.02
Men	77 (70-84)	75 (71-78)	78 (68-82)	.34	.003	.43	.004
Women	77 (71-83)	68 (68-69)	78 (71-86)	.005			
Time spent on hard exercise and strength training, h/wk	11 (8.5-17)	10 (5.0-14)	5.0 (4.0-10)	<.001	.44	<.001	.001
Men	11 (7-17)	10 (5.0-13.5)	5.0 (2.0-10)	<.001	.22	<.001	.02
Women	10 (10-17)	12 (6.0-17)	4.0 (4.0-7.0)	<.001	.62	<.001	.004
Hand grip strength, kg	58 (48-65)	51 (39-59)	53 (43-62)	.006	.02	.03	.74
Men	61 (55-70)	57 (50-61)	58 (51-64)	.006	.02	.03	.80
Women	40 (37-44)	35 (32-38)	40 (32-42)	.17			
Maximal bench press weight, kg	120 (100-153)	100 (60-120)	90 (78-110)	<.001	.003	<.001	.99
Men	140 (118-165)	110 (95-125)	100 (90-120)	<.001	.002	<.001	.77
Women	84 (50-95)	35 (30-60)	49 (30-60)	.049	.03	.07	.54
Family history of coronary disease	15 (19.5)	3 (12.0)	5 (8.8)	.20	NA	NA	NA
Men	8 (13.3)	3 (17.7)	2 (4.9)	.22	NA	NA	NA
Women	7 (41.2)	0	3 (18.8)	.07			
Cumulative lifetime total duration of AAS use, y	2.2 (1.2-7.2)	2.2 (1.0-5.5)	0 (0-0)	<.001	.36	<.001	<.001
Men	3.8 (1.3-9.8)	2.4 (1.2-5.5)	0 (0-0)	<.001	.44	<.001	<.001
Women	2.0 (0.5-2.8)	1.4 (0.4-3.9)	0 (0-0)	<.001	.38	<.001	<.001
Tobacco smoking status							
Total							
Never	32 (41.3)	12 (46.2)	39 (67.2)	.02	.43	.005	.20
Previous	21 (26.9)	9 (34.6)	12 (20.7)				
Current	25 (32.1)	5 (19.2)	7 (12.1)				
Men							
Never	25 (41.7)	8 (44.4)	29 (69.1)	.04	.01	.95	.12
Previous	16 (26.7)	5 (27.8)	9 (21.4)				
Current	19 (31.7)	5 (27.8)	4 (9.5)				
Women							
Never	7 (38.9)	4 (50.0)	10 (62.5)	.25	NA	NA	NA
Previous	5 (27.8)	4 (50.0)	3 (18.8)				
Current	6 (33.3)	0	3 (18.8)				
Weekly alcohol consumption, units^b^							
Total							
0	46 (59.0)	14 (52.9)	8 (13.8)	<.001	.77	<.001	.001
1-7	30 (38.5)	11 (42.3)	40 (69.0)				
8-14	2 (2.6)	1 (3.9)	10 (17.2)				
≥14	0	0	0				
Men							
0	36 (60.0)	8 (44.4)	5 (11.9)	<.001	.26	<.001	.02
1-7	23 (38.3)	9 (50.0)	27 (64.3)				
8-14	1 (1.7)	1 (5.6)	10 (23.8)				
≥14	0	0	0				
Women							
0	10 (55.6)	6 (75.0)	3 (18.8)	.02	.02	.77	.01
1-7	7 (38.9)	2 (25.0)	13 (81.3)				
8-14	1 (5.6)	0	0				
≥14	0	0	0				
Recreational drug use	15 (18.8)	5 (19.2)	0	<.001	.95	<.001	.002
Men	15 (24.6)	5 (27.8)	0	<.001	.77	<.001	.002
Women	0	0	0				

Abbreviations: AAS, androgenic anabolic steroids; BMI, body mass index (calculated as weight in kilograms divided by height in meters squared); NA, not applicable.

^a^ Variables were assessed in all participants (N = 164), men (n = 121 [73.8%]), and women (n = 43 [26.2%]). Values for continuous variables are shown as median (IQR) and for categorical variables as number (percentage) of persons. Group comparisons were performed using 1-way analysis of variance followed by Tukey honestly significant difference for post hoc analysis or the Kruskal-Wallis test followed by the Dunn test with Holm-Šídák adjustment for multiple comparisons, as appropriate. Post hoc tests were performed only when overall group differences were statistically significant.

^b^ One unit is defined as 12 g of pure alcohol.

Duration of AAS use ranged from 3.6 months to 42.0 years in active users and from 3.6 months to 24.5 years in previous users. Median cumulative lifetime duration of AAS intake for active users was 2.2 years (IQR, 1.2-7.2 years) and for previous users was 2.2 years (IQR, 1.0-5.5 years) (Table 1). However, men had a longer lifetime duration of AAS use than women, irrespective of whether they were active users or previous users (Table 1). Distribution of cumulative years of AAS use is illustrated in eFigure 1 in Supplement 1. Previous users reported discontinuing AAS intake a median of 2.5 years (range, 3 months to 29 years) before the study, with no difference between men and women. The type of AAS differed between men and women. Testosterone was the preferred choice among men (53 of 73 [72.6%]), whereas women primarily used oxandrolone (10 of 23 [43.5%]) (eFigure 2 in Supplement 1). Creatinine, human growth hormone, and weight-loss agents constituted the most frequently used substances alongside AAS (eFigure 3 in Supplement 1).

Systolic blood pressure was elevated in active AAS users compared with previous users and nonusers, while diastolic blood pressure was lowest in previous users (Table 1). However, more previous AAS users received antihypertensive medication compared with both active users and nonusers (eTable 1 in Supplement 1). In addition, AAS users had higher rates of mental disorders such as attention-deficit/hyperactivity disorder and anxiety compared with nonusers (eTable 1 in Supplement 1).

Regarding lifestyle, among active AAS users, there was a higher prevalence of current smokers and fewer never-smokers compared with nonusers (Table 1). In contrast, both active and previous AAS users reported significantly lower alcohol consumption than nonusers, whereas the use of recreational drugs was more common among AAS users than nonusers and was apparently limited to men.

### Biochemistry

Plasma hematocrit as well as creatinine and high-density lipoprotein levels differed among the groups, whereas neither low-density lipoprotein, glycated hemoglobin, average glucose, nor triglyceride levels differed. The metabolic findings were mostly consistent across sexes (eTable 2 in Supplement 1). When compared with their sex-matched groups of previous AAS users and nonusers, men currently using AAS had significantly lower levels of follicle-stimulating hormone (FSH), luteinizing hormone (LH), and sex hormone binding globulin (SHBG) but higher testosterone levels, whereas women currently using AAS had similar levels of FSH, LH, and testosterone but lower levels of SHBG (eTable 2 in Supplement 1).

### Atherosclerosis

Of the 18 men who previously used AAS, 1 (5.6%) reported a non–ST elevation myocardial infarction requiring stenting. No other participants had any history of atherosclerosis. No group differences were found for femoral (active users, 15 of 76 [19.7%]; previous users, 5 of 26 [19.2%]; nonusers, 11 of 53 [20.8%]; *P* = .89) or carotid (active users, 24 of 77 [31.2%]; previous users, 12 of 26 [46.2%]; nonusers, 13 of 54 [24.1%]; *P* = .47) artery plaques or CAC scores (median of 0 across all groups, with a range of 0-228 for active users [n = 80]; 0-800 for previous users [n = 26], and 0-163 for nonusers [n = 58]; *P* = .36) (Table 2), whereas a statistically significant difference in the prevalence of coronary NCPs was found between active users (19 of 80 [23.8%]) and nonusers (6 of 58 [10.3%]) (*P* = .03).

**Table 2.** Carotid and Femoral Artery Plaques by Ultrasonography and CAC Score by CT

Dependent variable^a^	Participants (N = 164)^b^			*P* value for group comparison
	Active AAS users	Previous AAS users	Nonusers	
Coronary NCPs	19/80 (23.8)	4/26 (15.4)	6/58 (10.3)	.04
CAC score, median (range)^c^	0 (0-228)	0 (0-800)	0 (0-163)	.36
Femoral artery plaque	15/76 (19.7)	5/26 (19.2)	11/53 (20.8)	.89
Carotid artery plaque	24/77 (31.2)	12/26 (46.2)	13/54 (24.1)	.47

Abbreviations: AAS, androgenic anabolic steroids; CAC, coronary artery calcium; CT, computed tomography; NCP, noncalcified plaque.

^a^ Results are from ultrasonography of the femoral artery and the carotid and coronary CT scans of the participants in the entire cohort stratified by AAS use.

^b^ Data are presented as number/total number (percentage) of participants unless otherwise indicated.

^c^ The CAC variable was dichotomized (CAC = 0 vs CAC > 0). Data are from 80 active users, 26 previous users, and 58 nonusers.

**Figure 1.**
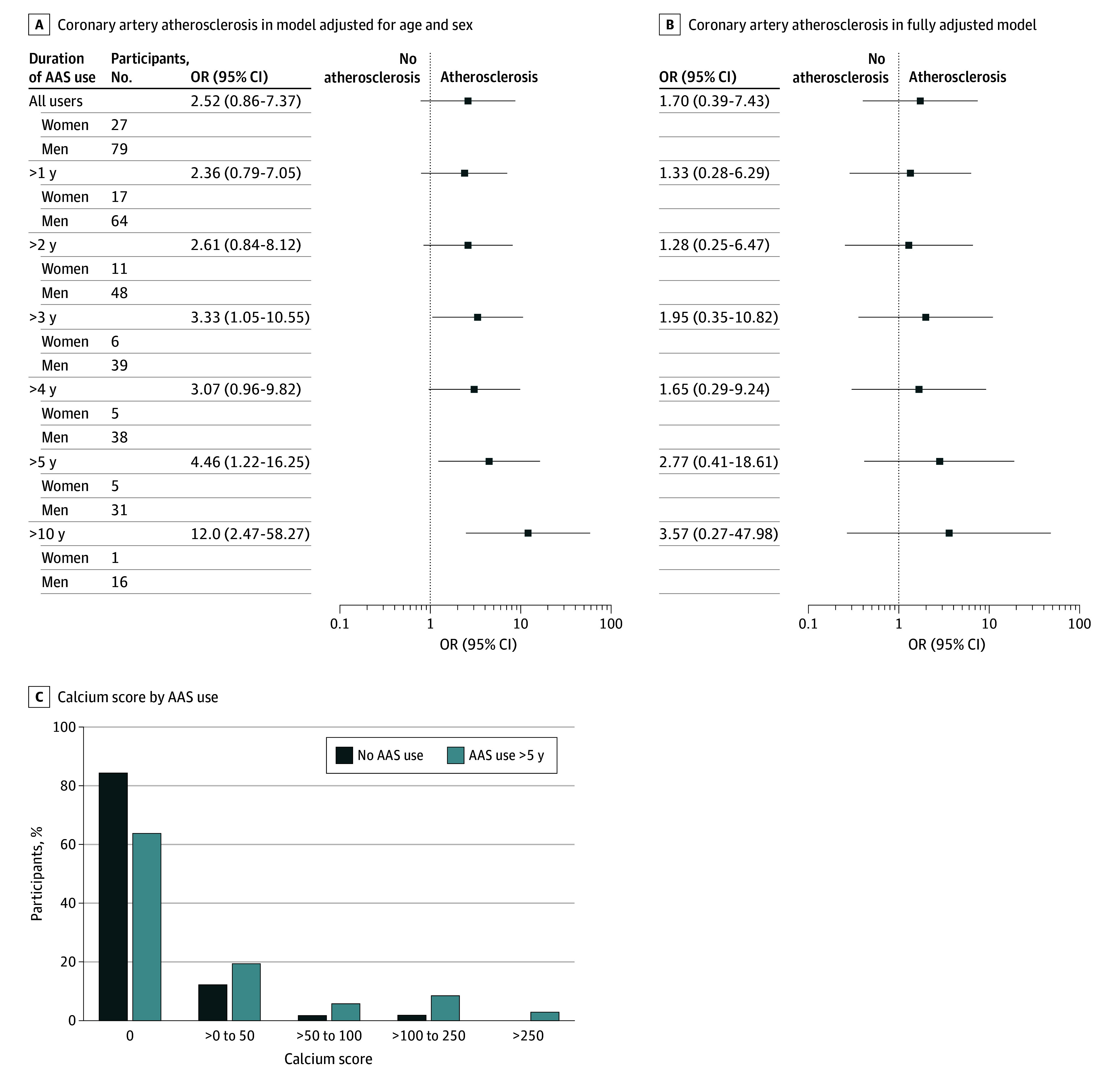
Coronary Artery Atherosclerosis and Calcified Plaque Severity by Lifetime Duration of Anabolic Androgenic Steroids (AAS) Use A, B, Logistic regression analysis compared the presence (coronary artery calcification [CAC] score >0) vs absence (CAC score = 0) of coronary artery atherosclerosis in previous and active AAS users (n = 106) vs nonusers (n = 58), stratified by cumulative lifetime duration of AAS intake. The fully adjusted model was adjusted for age, sex, body fat percentage, family history of coronary artery disease, blood pressure, total cholesterol, use of recreational drugs, tobacco smoking status, alcohol consumption, and hours of hard exercise and strength training per week. C, Thirty-six participants had AAS intake of more than 5 years and 58 were nonusers (n = 94; χ^2^ = 9.78; *P* = .04). OR indicates odds ratio.

We subsequently evaluated cumulative lifetime duration of AAS use as an independent factor associated with atherosclerosis. Both univariable and multivariable logistic regression analyses for presence of calcification (yes, no) revealed that cumulative AAS use was associated with a positive CAC score (multivariable OR, 1.23; 95% CI, 1.09-1.39; *P* = .001) and the presence of coronary NCPs (multivariable OR, 1.17; 95% CI, 1.05-1.30; *P* = .004), whereas AAS use was associated with carotid and femoral artery plaques only in the univariate model (Table 3). We subsequently stratified AAS users into subgroups based on durations of use (>1, >2, >3, >4, >5, and >10 years) (Figure 1A). After adjusting for age and sex, increased odds of a positive CAC score were associated with more than 3 years (OR, 3.33; 95% CI, 1.05-10.55), more than 5 years (OR, 4.46; 95% CI, 1.22-16.25), and more than 10 years (OR, 12.0; 95% CI, 2.47-58.27) of AAS use but not with more than 4 years (OR, 3.07; 95% CI, 0.96-9.82). No significant associations were observed in the fully adjusted model (Figure 1B).

**Table 3.** Atherosclerotic Stigmata and Their Association With Cumulative Lifetime Duration of AAS Use

Dependent variable	OR (95% CI) per unit increase in cumulative lifetime duration of AAS use, y^a^			
	Univariable logistic regression	*P* value	Multivariable logistic regression	*P* value
Coronary NCPs	1.17 (1.09-1.26)	<.001	1.17 (1.05-1.30)	.004
CAC score >0^b^	1.18 (1.09-1.27)	<.001	1.23 (1.09-1.39)	.001
Femoral artery plaque	1.05 (1.00-1.10)	.04	0.98 (0.92-1.05)	.63
Carotid artery plaque	1.10 (1.03-1.17)	.002	1.08 (0.99-1.18)	.09

Abbreviations: AAS, androgenic anabolic steroids; CAC, coronary artery calcium; NCP, noncalcified plaque; OR, odds ratio.

^a^ Univariable and multivariable logistic regression analyses of the entire population (N = 164) were performed with cumulative lifetime duration of AAS use in years as the independent variable for atherosclerotic measures. The multivariable model included the following covariables: age, body fat percentage, family history of coronary artery disease, blood pressure, total cholesterol, use of recreational drugs, tobacco smoking status, alcohol consumption, and hours of hard exercise and strength training per week.

^b^ The CAC variable was dichotomized (CAC = 0 vs CAC>0).

Logistic regression analysis did not account for calcification severity. Accordingly, we compared the 58 nonusers and the 36 users (34.0% of all users) with cumulative AAS use of more than 5 years according to CAC score (0, >0 to 50, >50 to 100, >100 to 250, and >250). The 2 groups did not differ with regard to median age (39 [IQR, 33-52] years for those with >5 years of AAS use vs 40 [IQR, 31-46] years for nonusers; *P* = .47) or sex distribution (31 men [86.1%] among those with >5 years of AAS use vs 42 men [72.4%] among nonusers; *P* = .12). The threshold of more than 5 years of AAS use was based on the logistic regression analysis. Using this approach, AAS users with a cumulative use of AAS exceeding 5 years had significantly more severe CAC scores as compared with nonusers (n = 94; χ^2^ = 9.78; *P* = .04) (Figure 1C).

### Echocardiography

Of the 61 male active AAS users, 1 (1.6%) was diagnosed with nonischemic congestive heart failure and received angiotensin-converting enzyme inhibitor and β-blocker medication. Nobody else had any history of structural heart disease.

Active and previous AAS users demonstrated numerous differences when compared with nonusers (eTable 3 in Supplement 1). A gradual decline in LVEF was found, with active AAS users showing the lowest values compared with previous AAS users and nonusers. The same pattern was evident for GLS, with cumulative AAS use associated with worsening LV GLS (regression coefficient, 0.08; 95% CI, 0.03-0.12; *P* = .002) and RV GLS (regression coefficient, 0.08; 95% CI: 0.03-0.13; *P* = .001). Moreover, when evaluating ventricular relaxation, active AAS users exhibited higher E/E′ ratios compared with previous users and nonusers. These echocardiographic findings were consistent across sexes (eFigure 4 in Supplement 1). In contrast, left atrial volume did not differ between active users, previous users, and nonusers (eTable 3 in Supplement 1).

**Figure 2.**
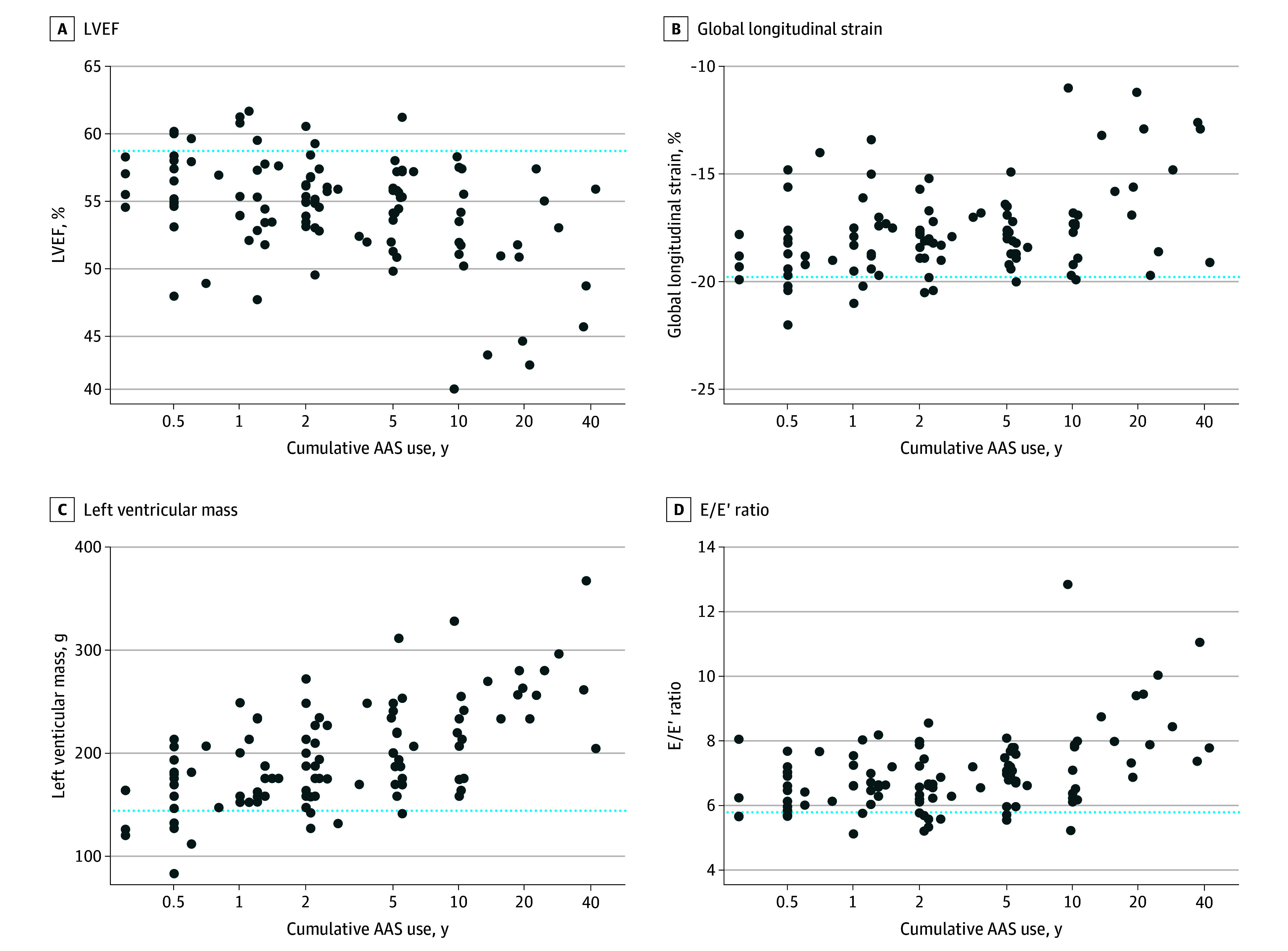
Associations Between Echocardiographic Parameters and Lifetime Duration of AAS Use The dotted blue lines represent the median of the normal range. AAS indicates androgenic anabolic steroids; E, early mitral peak velocity; E′, left ventricular relaxation; LVEF, left ventricular ejection fraction.

Multivariate analysis revealed several significant associations between cumulative lifetime AAS use and echocardiographic measures (eTable 4 in Supplement 1). Participants with prolonged AAS use exhibited more pronounced cardiac impairments. Figure 2 highlights associations between cumulative AAS intake and key echocardiographic parameters, showing that nearly all athletes with more than 5 years of AAS use had values outside the median of the normal range: LVEF (35 of 36 [97.2%]), LV mass (35 of 36 [97.2%]), E/E′ ratio (33 of 36 [91.7%]), and GLS (34 of 36 [94.4%]).

## Discussion

The cardiovascular consequences of illicit AAS use are primarily derived from case reports,^7,8,10,12,13^ minor observational studies,^9,29^ and epidemiological data,^6^ predominantly involving men in specific athletic cohorts. Therefore, this study aimed to provide insights into a broader population of AAS-using recreational athletes, including women. Data were successfully collected from 106 recreational athletes with a history of AAS use, including 27 women. Although the present study’s men-to-women ratio may be coincidental, it roughly aligns with estimates that AAS use is approximately 4 times less common in women than men.^3^

We observed a significant difference in the prevalence of coronary NCPs between active AAS users and nonusers (Table 2), but no significant differences as regards the prevalence of femoral or carotid artery plaques or CAC scores. This may be due to the inclusion of many young athletes with short-term AAS use. In exploratory regression analyses, cumulative lifetime AAS use was an independent factor associated with both calcified and noncalcified coronary artery plaques (Table 3). These findings should be interpreted with caution given the risk of type I error, and while the associations are hypothesis-generating and do not establish causality, they align with prior observations by Baggish et al,^15^ who found an association between lifetime AAS exposure and coronary atherosclerosis in experienced male weightlifters. Thus, further research is needed to clarify these associations.

The logistic regression analysis, adjusted for sex and age, revealed that lifetime duration of AAS use of more than 3 years, more than 5 years, and more than 10 years was significantly associated with an increased likelihood of a positive CAC score (Figure 1). When the model was fully adjusted for additional risk factors, there were no associations with duration of AAS use. However, the logistic regression analysis did not account for calcification severity. Therefore, we investigated the association between CAC severity and lifetime duration of AAS use (Figure 1). Based on the regression analysis, we used a cutoff of more than 5 years of cumulative AAS intake. Using this approach, cumulative AAS use for more than 5 years was associated with an increased CAC score. Again, our data align with findings by Baggish et al,^15^ thereby suggesting that findings in selected AAS-using athletes are applicable to a broader population of recreational athletes. However, these analyses are exploratory and should be interpreted as providing preliminary insight as well as highlighting the potential roles of both AAS exposure and broader lifestyle-related cardiovascular risk factors in recreational athletes who use AAS.

The observed declines in LV and RV systolic function, as well as LV diastolic function, suggest a global impact of AAS on cardiac function. This finding adds to previous research that predominantly focused on LV systolic function.^16,26,29^ Furthermore, the echocardiographic findings appeared to occur early in the course of AAS use. Of note, after more than 5 years of intake of AAS, virtually all AAS users had echocardiographic findings that were outside the median of the normal range (Figure 2). In conjunction with the CAC score, this indicates that 5 years of AAS exposure may constitute a threshold whereafter the odds of having increased coronary calcification and subnormal echocardiography results are significantly increased compared with no AAS use. On the other hand, our data showed that myocardial dysfunction was less prevalent in previous AAS users compared with active users, suggesting that discontinuation can reduce some of the cardiac AAS-related changes.

As regards lifestyle factors (Table 1), active and previous AAS users of both sexes reported lower alcohol intake compared with nonusers. Conversely, the use of recreational drugs (primarily cocaine and cannabis), both established cardiovascular risk factors,^34,35,36^ was reported exclusively by men, indicating a potential difference in risk behavior between male and female AAS users.

AAS use was associated with the expected endocrine changes in men (eTable 2 in Supplement 1).^37^ In contrast, women using AAS had normal FSH, LH, testosterone, and estrogen levels but suppressed SHBG. The reason for this discrepancy remains unknown. Nevertheless, urinary measurements confirmed that both men and women with active AAS use were accurately categorized.

### Limitations

This study has limitations. The cross-sectional study design excludes causal conclusions regarding our findings. The exploratory analyses offer preliminary insight into the potential roles of AAS exposure and lifestyle-related cardiovascular risk factors in the study population. In addition, due to the risk of selection bias, it cannot be concluded that the cohort fully represents the broader population of Danish AAS-using recreational athletes. However, our observed men-to-women ratio in AAS users aligned with existing estimates.^3^ We also acknowledge that data on accumulated lifetime use of AAS are likely to contain recall bias.

## Conclusions

In this cross-sectional study, cumulative lifetime AAS intake in recreational male and female athletes in Denmark was independently associated with both soft and hard coronary plaques, and our data suggested that a cumulative use of AAS exceeding 5 years constituted a threshold for increased coronary calcification. Furthermore, we found associations between the accumulated use of AAS and myocardial changes on echocardiography. Thus, preventive measures against the use of AAS in recreational sports should target both men and women

## Notes

Supplement 1. **eTable 1.** Medical Conditions and Prescribed Medication Use Derived From Medical Records **eTable 2.** Biochemical Characteristics of the Participants in the Entire Cohort Stratified by AAS Use **eTable 3.** Echocardiographic Findings **eTable 4.** Univariable and Multivariable Linear Regression Analyses of the Entire Population With Cumulative Lifetime Duration of AAS Use as an Independent Predictor for Echocardiographic Characteristics **eFigure 1.** Cumulative Years of AAS Use in Active and Previous AAS Users **eFigure 2.** Type of AAS at Maximum Dose **eFigure 3.** Other Substances Used Alongside AAS in Active and Previous AAS Users **eFigure 4.** Primary Echocardiographic Measures in Men and Women Stratified by AAS Use

Supplement 2.
Data Sharing Statement
