# Supplementary material for: Illicit Anabolic Steroid Use and Cardiovascular Status in Men and Women
Source: JAMA Netw Open. 2025 Aug 29;8(8):e2526636. doi: 10.1001/jamanetworkopen.2025.26636 (PMC12397887; doi:10.1001/jamanetworkopen.2025.26636)
Supplement: Supplement 1. — eTable 1. Medical Conditions and Prescribed Medication Use Derived From Medical Records eTable 2. Biochemical Characteristics of the Participants in the Entire Cohort Stratified by AAS Use eTable 3. Echocardiographic Findings eTable 4. Univariable and Multivariable Linear Regression Analyses of the Entire Population With Cumulative Lifetime Duration of AAS Use as an Independent Predictor for Echocardiographic Characteristics eFigure 1. Cumulative Years of AAS Use in Active and Previous AAS Users eFigure 2. Type of AAS at Maximum Dose eFigure 3. Other Substances Used Alongside AAS in Active and Previous AAS Users eFigure 4. Primary Echocardiographic Measures in Men and Women Stratified by AAS Use [file jamanetwopen-e2526636-s001.pdf]

Supplementary Online Content

Buhl LF, Christensen LL, Hjortebjerg R, et al. Illicit anabolic steroid use and cardiovascular status in men and women. *JAMA Netw Open*. 2025;8(8):e2526636. doi:10.1001/jamanetworkopen.2025.26636

eTable 1. Medical Conditions and Prescribed Medication Use Derived From Medical Records
eTable 2. Biochemical Characteristics of the Participants in the Entire Cohort Stratified by AAS Use
eTable 3. Echocardiographic Findings
eTable 4. Univariable and Multivariable Linear Regression Analyses of the Entire Population With Cumulative Lifetime Duration of AAS Use as an Independent Predictor for Echocardiographic Characteristics
eFigure 1. Cumulative Years of AAS Use in Active and Previous AAS Users
eFigure 2. Type of AAS at Maximum Dose
eFigure 3. Other Substances Used Alongside AAS in Active and Previous AAS Users
eFigure 4. Primary Echocardiographic Measures in Men and Women Stratified by AAS Use

This supplementary material has been provided by the authors to give readers additional information about their work.

© 2025 Buhl LF et al. *JAMA Network Open*.

eTable 1

Medical conditions and prescribed medication use derived from medical records

Categorical variables are indicated as number (n) or percentage (%) of persons.

AAS, androgenic anabolic steroids; ADHD, attention deficit hyperactivity disorder; n.d., not determined.

*: P <.05 compared with non-users; **: P <.05 compared with previous users.

**Medical condition, %**	**Active AAS users (n=80), %**	**Previous AAS users (n=26), %**	**Non-users (n=58), %**	**P value**
ADHD	11.1	22.7*	3.6	.036
Anxiety	36.0*	46.2*	12.3	.001
Depression	32.9*	50.0*	8.9	.000
Eating disorder	11.4	13.0	3.6	.22
Asperger's syndrome	1.4	4.4	0.0	.31
Stroke	2.6	8.0	0.0	.11
Heart attack	1.3	4.2	0.0	.32
Deep vein thrombosis	1.3	8.7	3.5	.21
Aortic aneurism	0.0	0.0	0.0	n.d.
Hypertension	32.5*	16.7	8.8	.004
Diabetes	1.3	0.0	0.0	.59
Hypercholesterolemia	26.3	20.8	10.7	.084

**Medication, n (%)**	**Active AAS users (n=80), %**	**Previous AAS users (n=26), %**	**Non-users (n=58), %**	**P value**
Antidepressants	8.8	7.7	1.7	.22
Antipsychotics	2.5	0.0	1.7	.71
Antihypertensive medications	7.5**	26.9*	1.7	.001
Diuretics	2.5	3.9	0.0	.39
Statins	2.5	3.9	5.2	.71
Analgesics	5.0	7.7	0.0	.15

© 2025 Buhl LF et al. *JAMA Network Open*.

eTable 2

Biochemical characteristics of the participants in the entire cohort stratified by AAS use

Values for continuous variables are shown as median (interquartile range). AAS, androgenic anabolic steroids; FSH, follicle-stimulating hormone; HbA1c, glycated hemoglobin; HDL, high-density lipoprotein; IQR, interquartile range; LDL, low-density lipoprotein; LH, luteinizing hormone; SHBG, sex hormone binding globulin.

Variable	Active AAS users (n=80), median (IQR)	Previous AAS users (n=26), median (IQR)	Non-users (n=58), median (IQR)	P value$_{groups}$	P value Active AAS users vs. previous users	P value Active AAS users vs. non-users	P value Previous AAS users vs. non-users
Hematocrit, %	47 (44;51)	43 (40;46)	41 (39;42)	<.001	<.001	<.001	.026
- Men	48 (46;51)	44 (42;47)	42 (41;43)	<.001	<.001	<.001	.019
- Women	43 (41;45)	41 (37;42)	37 (35;39)	<.001	.021	<.001	.17
Creatinine, µmol/L	102 (90;111)	87 (79;102)	82 (71;88)	<.001	.015	<.001	.042
- Men	105 (92;112)	95 (85;103)	84 (80;98)	<.001	.07	<.001	.12
- Women	90 (77;105)	75 (73;86)	68 (65;73)	<.001	.21	<.001	.08
HbA1c, mmol/mol	33 (31;34)	33 (31;34)	34 (32;36)	.31			
- Men	32 (30;34)	33 (30;34)	34 (32;36)	.09			
- Women	34 (32;35)	33 (32;35)	32 (31;35)	.56			
Total cholesterol, mmol/L	4.1 (3.6;4.7)	4.4 (3.8;5.1)	4.7 (3.9;5.1)	.042	.53	.033	.69
- Men	4.2 (3.6;4.7)	4.2 (3.4;4.8)	4.7 (3.9;5.1)	.10			
- Women	4.1 (3.4;4.4)	4.9 (4.2;5.3)	4.8 (3.9;5.1)	.029	.031	.18	.49
HDL cholesterol, mmol/L	0.9 (0.7;1.1)	1.4 (1.1;1.6)	1.4 (1.2;1.7)	<.001	<.001	<.001	.52
- Men	0.9 (0.7;1.1)	1.3 (1.0;1.4)	1.3 (1.1;1.7)	<.001	<.001	<.001	.32
- Women	0.8 (0.7;1.1)	1.7 (1.5;1.9)	1.7 (1.5;1.8)	<.001	<.001	<.001	.99
LDL cholesterol, mmol/L	2.6 (2.2;3.3)	2.2 (1.8;2.7)	2.3 (2.0;2.9)	.15			
- Men	2.7 (2.1;3.3)	2.1 (1.7;2.6)	2.3 (1.9;2.9)	.07			
- Women	2.5 (2.2;3.5)	2.5 (2.1;3.3)	2.7 (2.1;2.9)	.68			
Triglycerides, mmol/L	1.2 (0.8;2.0)	1.2 (0.8;2.1)	1.1 (0.8;2.1)	.89			
- Men	1.2 (1.0;2.0)	1.2 (0.8;2.2)	1.5 (0.9;2.4)	.52			
- Women	0.7 (0.6;1.4)	1.1 (0.8;1.5)	0.9 (0.6;1.1)	.36			
Estradiol (nmol/L)	0.12 (0.05;0.27)	0.06 (0.04;0.09)	0.08 (0.05;0.11)	.002	.003	.012	.12
- Men	0.12 (0.06;0.28)	0.05 (0.04;0.07)	0.06 (0.05;0.09)	<.001	<.001	<.001	.23
- Women	0.10 (0.04;0.23)	0.07 (0.04;0.28)	0.15 (0.09;0.36)	.61			
FSH (IU/L)	<0.3 (<0.3;1.5)	4.5 (2.0;7.0)	5.5 (3.7;8.1)	<.001	<.001	<.001	.08

© 2025 Buhl LF et al. *JAMA Network Open*.

- Men	<0.3 (<0.3;<0.3)	3.5 (1.9;6.5)	5.5 (3.7;8.1)	<.001	<.001	<.001	.035
- Women	4.4 (1.3;7.0)	6.6 (3.6;37)	5.4 (3.4;7.8)	.38			
LH (IU/L)	<0.3 (<0.3;1.6)	5.4 (4.0;8.3)	5.4 (4.1;8.7)	<.001	<.001	<.001	.31
- Men	<0.3 (<0.3;<0.3)	5.3 (4.3;6.9)	5.2 (4.1;7.5)	<.001	<.001	<.001	.26
- Women	2.8 (<0.3;5.0)	8.4 (2.6;27)	7.9 (3.8;11)	.12			
Testosterone (nmol/L)	28 (5.9;47)	10 (1.0;15)	12 (1.1;15)	<.001	.001	<.001	.46
- Men	34 (19;58)	14 (10;17)	14 (11;16)	<.001	<.001	<.001	.46
- Women	0.3 (0.2;7)	0.8 (0.6;1.0)	0.6 (0.4;0.9)	.38			
SHBG (nmol/L)	12 (7;22)	47 (28;61)	39 (30;58)	<.001	<.001	<.001	.86
- Men	12 (7;23)	40 (23;50)	35 (28;44)	<.001	<.001	<.001	.96
- Women	12 (6;22)	71 (50;122)	79 (56;99)	<.001	<.001	<.001	.96

© 2025 Buhl LF et al. *JAMA Network Open*.

eTable 3

Echocardiographic findings

Echocardiographic characteristics of the participants in the entire cohort stratified by AAS use. Values for continuous variables are shown as median (interquartile range). Categorical variables are indicated as number (n) and percentage (%) of persons. AAS, androgenic anabolic steroids; IQR, interquartile range.

Variable	Active AAS users (n=80), median (IQR)	Previous AAS users (n=26), median (IQR)	Non-users (n=58), median (IQR)	P value$_{groups}$	P value Active AAS users vs. previous users	P value Active AAS users vs. non-users	P value Previous AAS users vs. non-users
Left ventricular ejection fraction, %	54 (52;56)	57 (55;58)	59 (56;61)	<.001	.001	<.001	.027
Left ventricular global longitudinal strain, (%)	-18 (-19;-17)	-19 (-20;-18)	-20 (-21;-19)	<.001	.002	<.001	.047
Right ventricular global longitudinal strain, (%)	-20 (21;-20)	-21 (-22;-21)	-23 (-24;-21)	<.001	.009	<.001	.001
Early mitral peak velocity, cm/s	72 (68;77)	71 (64;78)	74 (68;85)	.13			
Early left ventricular relaxation (septal) E', cm/s	9 (9;10)	10 (9;11)	12 (11;13)	<.001	.15	<.001	<.001
Early left ventricular relaxation (lateral) E', cm/s	12 (11;13)	13 (12;14)	15 (14;16)	<.001	.21	<.001	<.001
Early mitral peak velocity and average left ventricular relaxation (E/E') ratio	7.0 (6.3;7.8)	6.4 (5.7;6.8)	5.8 (5.1;6.3)	<.001	.005	<.001	.005
Left ventricular mass, g	194 (164;230)	176 (153;234)	144 (123;170)	<.001	0.13	<.001	.001
Left ventricular mass/body surface area, g/m2	93 (85;105)	85 (77;111)	68 (62;77)	<.001	.32	<.001	<.001
Relative wall thickness	0.42 (0.38;0.45)	0.41 (0.37;0.43)	0.36 (0.32;0.39)	<.001	.34	<.001	.002
Left atrial volume, ml	43 (40;47)	42 (38;47)	44 (41;49)	.19			
Cardiac remodeling and hypertrophy, n (%)<br>Normal geometry<br>Remodeling<br>Hypertrophy	 40 (50.0)<br>28 (35.0)<br>12 (15.0)	 16 (61.5)<br>6 (23.1)<br>4 (15.4)	 51 (87.9)<br>7 (12.1)<br>0 (0.0)	 <.001	 .51	 <.001	 .003

© 2025 Buhl LF et al. *JAMA Network Open*.

eTable 4

Univariable and multivariable linear regression analyses of the entire population with cumulative lifetime duration of AAS use as an independent predictor for echocardiographic characteristics

The multivariable model included co-variables age, sex, body fat percentage, family history of coronary artery disease, use of recreational drugs, tobacco use, alcohol consumption, and hours of hard exercise and strength training. AAS, androgenic anabolic steroids; CI, confidence interval.

	Cumulative lifetime duration of AAS use, years			
**Dependent variable**	**Univariable linear regression coefficient (95% CI)**	**P value**	**Multivariable linear regression coefficient (95% CI)**	**P value**
Left ventricular ejection fraction, %	-0.28 (-0.36;-0.19)	<.001	-0.16 (-0.27;0.6)	.001
Left ventricular global longitudinal strain	0.13 (0.09;0.17)	<.001	0.08 (0.03;0.12)	.002
Right ventricular global longitudinal strain	0.14 (0.10;0.18)	<.001	0.08 (0.03;0.13)	.001
Early mitral peak velocity, cm/s	-0.22 (-0.46;0.02)	.08	0.08 (-0.19;0.36)	.56
Early left ventricular relaxation (septal) E’, cm/s	-0.15 (-0.18;-0.11)	<.001	-0.06 (-0.10;-0.03)	<.001
Early left ventricular relaxation (lateral) E’, cm/s	-0.18 (-0.22;-0.14)	<.001	-0.08 (-0.12;-0.03)	<.001
Early mitral peak velocity and average left ventricular relaxation (E/E’) ratio	0.09 (0.07;0.12)	<.001	0.07 (0.04;0.09)	<.001
Left ventricular mass, g	4.3 (3.4;5.2)	<.001	2.5 (1.6;3.4)	<.001
Left ventricular mass/body surface area, g/m2	1.7 (1.3;2.1)	<.001	0.9 (0.5;1.3)	<.001
Relative wall thickness	0.004 (0.003;0.005)	<.001	0.002 (0.001;0.003)	.003
Left atrial volume, ml	0.25 (0.12;0.37)	.10	0.14 (0.02;0.26)	.22

© 2025 Buhl LF et al. *JAMA Network Open*.

eFigure 1

Cumulative years of AAS use in active and previous AAS users

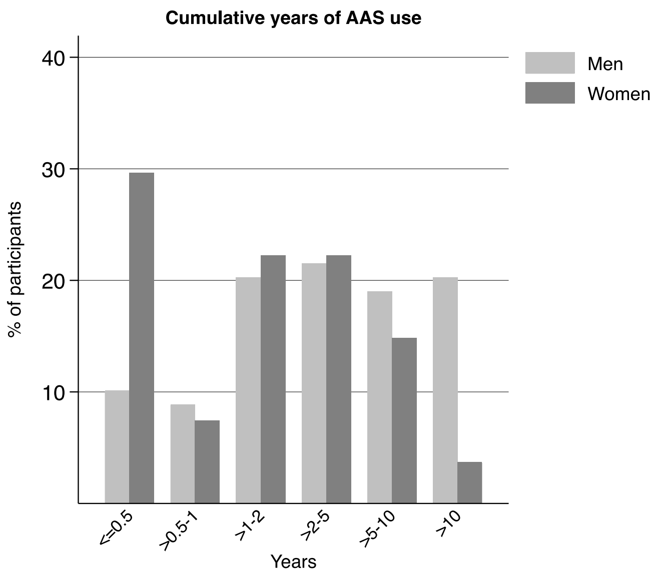

© 2025 Buhl LF et al. *JAMA Network Open*.

eFigure 2

Type of AAS at maximum dose

Types of AAS used by active and previous AAS users at the maximum dose in men (left panel) and women (right panel). AAS, androgenic anabolic steroids.

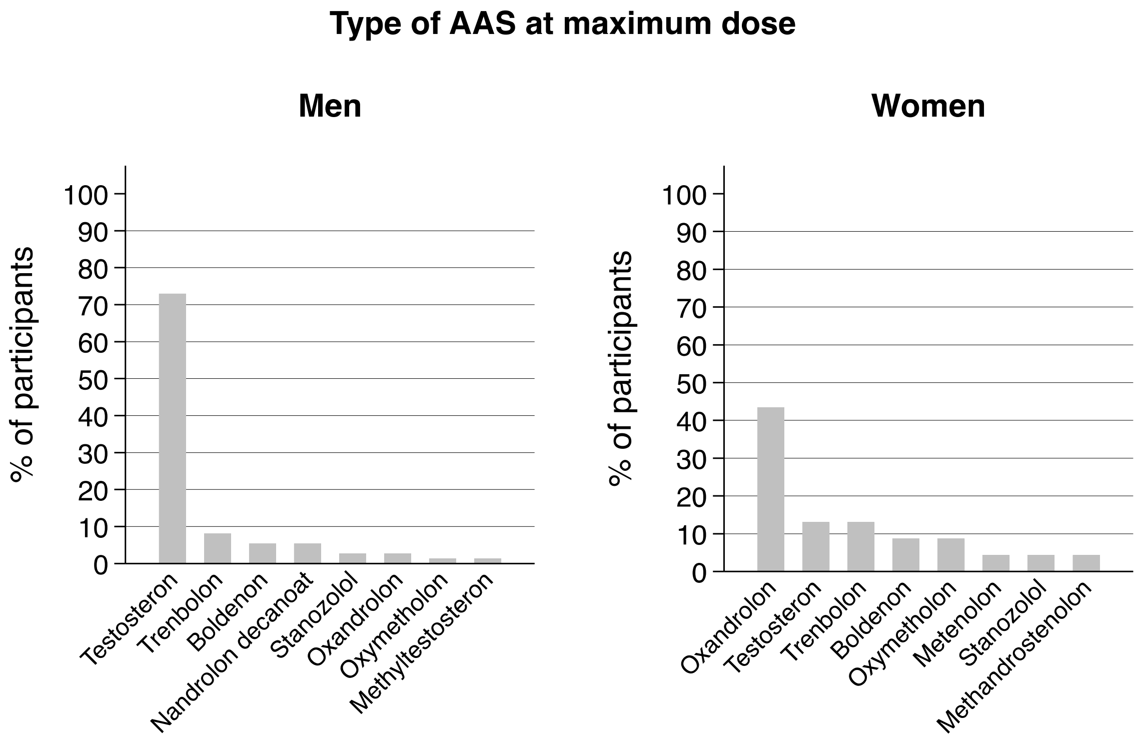

© 2025 Buhl LF et al. *JAMA Network Open*.

eFigure 3

Other substances used alongside AAS in active and previous AAS users

EPO, erythropoietin; GHRP, Growth hormone-releasing peptide; IGF-1, insulin-like growth factor-1; SARM, selective androgen receptor modulator; SERM, selective estrogen receptor modulator.

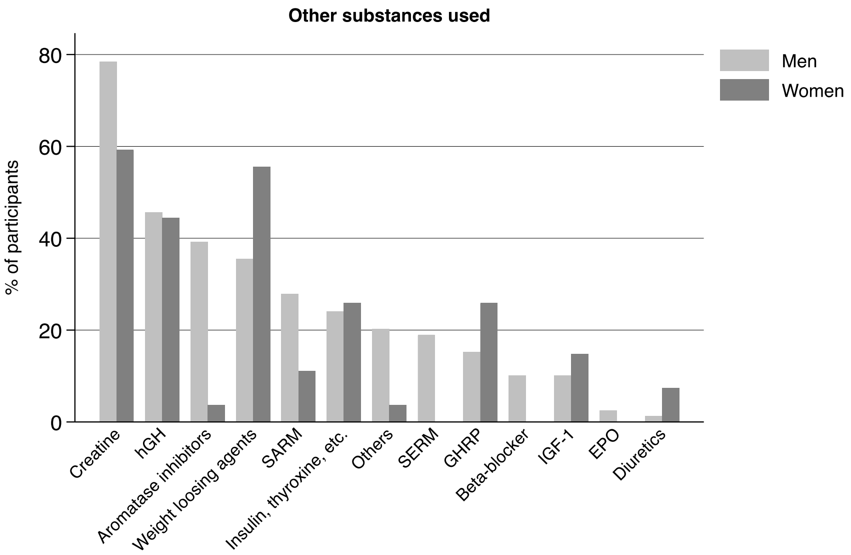

© 2025 Buhl LF et al. *JAMA Network Open*.

eFigure 4

Primary echocardiographic measures in men and women stratified by AAS use

A) LVEF, B) GLS, C) LV mass, and D) E/E' average. P-values indicate significant group differences by ANOVA within each sex.

AAS, androgenic anabolic steroids; E/E', early mitral peak velocity and average left ventricular relaxation ratio; GLS, global longitudinal strain; LVEF, left ventricular ejection fraction; LV, left ventricular.

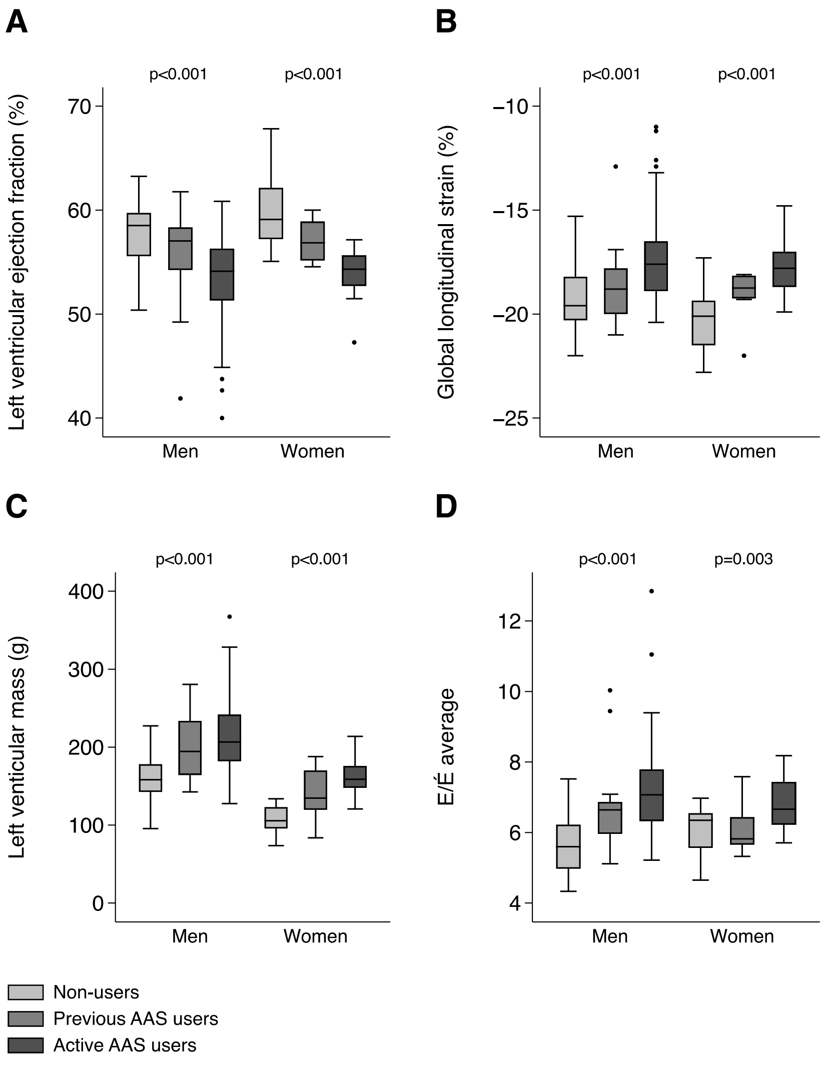

© 2025 Buhl LF et al. *JAMA Network Open*.
