# Supplementary material for: Illicit Anabolic Steroid Use and Cardiovascular Status in Men and Women
Source: JAMA Netw Open. 2025 Aug 29;8(8):e2526636. doi: 10.1001/jamanetworkopen.2025.26636 (PMC12397887; doi:10.1001/jamanetworkopen.2025.26636)
Supplement: Supplement 2. — Data Sharing Statement [file jamanetwopen-e2526636-s002.pdf]

Data Sharing Statement

Buhl. Illicit Anabolic Steroid Use and Cardiovascular Status in Men and Women. *JAMA Netw Open*. Published August 29, 2025. doi:10.1001/jamanetworkopen.2025.26636

Data
Data available: No

Additional Information
Explanation for why data not available: The data collected for this study, including individual patient data and a data dictionary, will not be made available due to the following reasons. The data include sensitive health information, and sharing could compromise participant privacy, even with de-identification. Our study protocols and ethical approvals do not include provisions for data sharing with external parties. Local data protection laws and regulations, such as GDPR, limit the sharing of personal health information collected during this study. We are committed to transparency and encourage collaboration. Researchers interested in our findings are welcome to contact us to discuss potential data access under controlled and compliant conditions.
